# Supplementary material for: Cross-modality synthesis of EM time series and live fluorescence imaging
Source: eLife. 2022 Jun 6;11:e77918. doi: 10.7554/eLife.77918 (PMC9213002; doi:10.7554/eLife.77918)
Supplement: Supplementary file 1. [file elife-77918-supp1.docx]

**Supplemental File 1:**

**S1a** EM Imaging Details

| **Stage** | **Estimated Time pfc** | **Slice Axial Orientation** | **Acquisition modality** | **Number of Sections** | **Lateral Resolution (nm)** | **Section Thickness (nm)** |
| --- | --- | --- | --- | --- | --- | --- |
| Bean | 320 min | ventral-dorsal | FIB | 944 | 9.9 | 30 |
| Comma | 345 min | anterior-posterior | FIB | 1641 | 8.5 | 25 |
| 1.5-fold | 380 min | left-right | AT | 325 | 9.7 | 65 |
| 2-fold | 475 min | ventral-dorsal | AT | 310 | 19.4 | 85 |

**S1b** EM Data Detailed Information

|  | **Bean stage** | **Comma stage** | **1.5-fold stage** |
| --- | --- | --- | --- |
| **Landmark Properties** | | | |
| **# Cells** | 576 | 578 | 601 |
| **Annotation Extent** | | | |
| **# Cells Confirmed Identity** | 148 | 231 | 242 |
| **# Cells Confirmed Tissue** | 198 | 177 | 95 |
| **Accuracy** | | | |
| **Single Cell Accuracy** | .78 | .73 | .71 |
| **Tissue Level Accuracy** | .95 | .91 | .79 |

**S1c** FM Data Detailed Information

|  | **Bean stage** | **Comma stage** | **1.5-fold stage** |
| --- | --- | --- | --- |
| **Landmark Properties** | | | |
| **# Adjacencies Observed** | 6170 | 5838 | 5881 |
| **# Consistent Adjacencies** | 439 | 504 | 501 |
| **# Inconsistent LM** | 28 | 27 | 34 |

**S1d** Details of FM Series Used to Time Neurite Initial Outgrowths

| **Cell** | **EM time** | **FM time** | **Strain Source**** | **Marker** | **Strain** | **Strain Genotype** |
| --- | --- | --- | --- | --- | --- | --- |
| AFD | 345 | 345 | [^29^](#_ENREF_29) | *egl-13* | DCR6358 | olaEx3765 [DACR2605(egl-13p::egl-13(168bp)::SL2::PHD::unc-54UTR) at 50ng/µl + DACR2603(sdz-31p::GFP::NLS::unc-54UTR) at 25ng/µl + DACR218(unc-122p::RFP) at 25ng/µl]; ujIs113 [pie1p::mCherry::H2B::pie-1 3'UTR + nhr-2p::his-24::mCherry::let-858 3'UTR + unc-119(+)] II |
| AIY | 345 | 324 | [^29^](#_ENREF_29) | *ttx-3* | DCR7844 | mgIs18 [ttx-3p::GFP] IV; olaEx4765 [DACR3254(ceh-24p::mCherry::unc-54UTR) at 50ng/uL + DACR218(unc-122p::RFP) at 30 ng/uL] |
| ALA | 345 | 347 | [^46^](#_ENREF_46) | *ceh-10* | BV395 | lqIs4 [ceh-10 promoter::GFP + rol-6]; ujIs113; olaEx2383[pMM23::pLim-4_PHD_Super_Folder_GFP_unc54 UTR in pDEST-R4-R3, pUnc-122::RFP] |
| ASH/RIB | 320 | 318 | [^46^](#_ENREF_46) | *cnd-1* | BV293 | zbIs3[cnd-1>PH::GFP] IV; ujIs113 II. |
| AVD | 345 | 339 | [^46^](#_ENREF_46) | *zag-1* | BV450 | ujIs113 II; ex?(zag-1:GFP, pUnc-122::RFP) |
| AVG | 345 | 347 | [^46^](#_ENREF_46) | *unc-86* | BV298 | kyIs262 [unc-86::myr GFP + odr-1::RFP] IV; ujIs113 II. |
| AWC | 320 | 324 | [^29^](#_ENREF_29) | *ceh-37* | DCR6298 | olaEx3716 [DACR2541(ceh-37p::ceh-37(103bp)::SL2::PHD::GFP::unc-54UTR) at 100ng/ul + DACR218(unc-122p::RFP) at 50ng/ul]; ujIs113 [pie-1p::mCherry::H2B::pie-1 3'UTR + nhr-2p::his24::mCherry::let-858 3'UTR + unc-119(+)] II |
| RIH | 345 | 350 | [^46^](#_ENREF_46) | *unc-86* | BV298 | kyIs262 [unc-86::myr GFP + odr-1::RFP] IV; ujIs113 II. |
| RIP | 345 | 330 | [^46^](#_ENREF_46) | *unc-86* | BV298 | kyIs262 [unc-86::myr GFP + odr-1::RFP] IV; ujIs113 II. |
| RIV+  * | 345 | 338 | [^46^](#_ENREF_46) | *lim-4* | BV286 | ujIs113; olaEx2383[pMM23::pLim-4_PHD_Super_Folder_GFP_unc54 UTR in pDEST-R4-R3, pUnc-122::RFP] |
| RMDD | 345 | 313 | [^46^](#_ENREF_46) | *zag-1* | BV450 | ujIs113 II; ex?(zag-1:GFP, pUnc-122::RFP) |
| RMED | 345 | 362 | [^46^](#_ENREF_46) | *ceh-10* | BV395 | lqIs4 [ceh-10 promoter::GFP + rol-6]; ujIs113; olaEx2383[pMM23::pLim-4_PHD_Super_Folder_GFP_unc54 UTR in pDEST-R4-R3, pUnc-122::RFP] |
| SAAV | 345 | 324 | [^46^](#_ENREF_46) | *lim-4* | BV286 | ujIs113; olaEx2383[pMM23::pLim-4_PHD_Super_Folder_GFP_unc54 UTR in pDEST-R4-R3, pUnc-122::RFP] |
| SIBD | 345 | 325 | [^46^](#_ENREF_46) | *lim-4* | BV286 | ujIs113; olaEx2383[pMM23::pLim-4_PHD_Super_Folder_GFP_unc54 UTR in pDEST-R4-R3, pUnc-122::RFP] |
| SMDD+ * | 320 | 297 | [^46^](#_ENREF_46) | *lim-4* | BV286 | ujIs113; olaEx2383[pMM23::pLim-4_PHD_Super_Folder_GFP_unc54 UTR in pDEST-R4-R3, pUnc-122::RFP] |

*Note that entries suffixed with a + are small clusters of cells named after a prominent member while all other entries are individually identified cells which are either, in general, spatially isolated in a given marker, or isolated in a particular data set due to mosaicism.

** Strain Sources

46 Barnes, K. *Spatial and Temporal Organization of Brain Morphogenesis at Single-Cell Resolution a Study in C. elegans* PhD thesis, Weill Cornell Graduate School of Medical SciencesWeill Cornell, (2020).

29 Moyle, M. W. *et al.* Structural and developmental principles of neuropil assembly in C. elegans. *Nature* **591**, 99-104, doi:10.1038/s41586-020-03169-5 (2021).

**S1e** Individual Cell Annotation Summary

| **Tissue** | **Subtype** | **Bean stage** | **Comma Stage** | **1.5 Stage** |
| --- | --- | --- | --- | --- |
| **Neurons** | Neurons- general | 46 | 67 | 63 |
|  | Amphid | 11 | 25 | 10 |
|  | Support cells | 15 | 12 | 9 |
| **Epidermal** | Epidermal-general | 34 | 23 | 23 |
|  | Seam cells | 20 | 19 | 20 |
| **Muscle** |  | 1 | 38 | 75 |
| **Pharynx** |  | 2 | 0 | 1 |
| **Gut** |  | 5 | 20 | 20 |
| **Other** |  | 14 | 27 | 21 |
| **# Cells Confirmed Identity Total** | | **148** | **231** | **242** |

Note: Consult Supplemental Files and WebKnossos for a full list of individual annotated confirmed cell names.

**S1f** Additional Alignment Accuracy Assessment

|  | **Bean** | **Comma** | **1.5-fold** |
| --- | --- | --- | --- |
| **EM time Series** | .78 | .73 | .71 |
| **Additional Confocal FM 1** | 0.69 | 0.72 | 0.74 |
| **Additional Confocal FM 2** | 0.74 | 0.65 | 0.62 |
| **Additional Confocal FM 3** | 0.82 | 0.70 | 0.72 |

**S1g** Landmarks Used to Initialize Alignment

| **Cells** | **Bean stage** | **Comma stage** | **1.5-fold stage** | **Purpose** |
| --- | --- | --- | --- | --- |
| Seam Cells | All | All | All | Provide A-P body axis and approximate twist of L-R axis along A-P. |
| Gut Cells | All | None | All |  |
| Body Wall Muscles | None | None | All |  |
| Distinctively positioned/shaped neurons: | RIH, RMDDL/R, AVL | PVQL/R | RIH, RMDDL/R, AVL,  ALA | Refine alignment of ganglia in head and major cell clusters in tail |
| Other distinctively positioned cells: | exc_cell, p11,12,  spike l/r | exc_cell, p11,12 | exc_cell,  z4, z1 |  |
| Distinctive Anterior Epidermal Cells: | hyp1 (ABarappaapa) hyp3(ABplaapaaaa)  hyp4 (ABarpapapa) | hyp4 x3  (ABarpapapa, ABpraappaa, ABplaappaa) | hyp4 x2 (ABpraappaa, ABplaappaa) | Mark anterior of head |
|  | hyp6 x2  (ABplaaaapa, ABarpaapaa) | hyp6 x4  (ABplaaaapa, ABarpaapaa, ABplaaaapp, ABarpapapp) | hyp6 x2  (ABplaappap, ABpraappap) | Refine L-R axis in head |
|  |  | hyp7 x3  (ABarpaapap,  ABplaapppp, ABpraapppp) | hyp7 x4 (ABpraapppa, ABplaapppa, ABplaapppp, ABpraapppp) | Refine L-R axis in head |
| Distinctive Posterior Epidermal Cells: | hyp7 x2  (Caappd, Cpappd) |  |  | Refine L-R axis in tail |
|  | hyp8,9 |  | hyp8,9 | Mark posterior of tail |
